# Supplementary material for: Tuning of the Titanium Oxide Surface to Control Magnetic Properties of Thin Iron Films
Source: Materials (Basel). 2022 Dec 28;16(1):289. doi: 10.3390/ma16010289 (PMC9822368; doi:10.3390/ma16010289)
Supplement: Supplementary file 1 [file materials-16-00289-s001.zip › materials-2074630-supplementary.pdf]

Supplementary Materials

Figure S1 shows magnetization curve and derivative of upper branch of *M(H)* for sample S50 before (A) and after (B) heat treatment. Measurements were carried out in the in-plane geometry. Hysteresis loops were fitted according to the T(x) model for the case of two independent magnetic phases ($n = 2$).

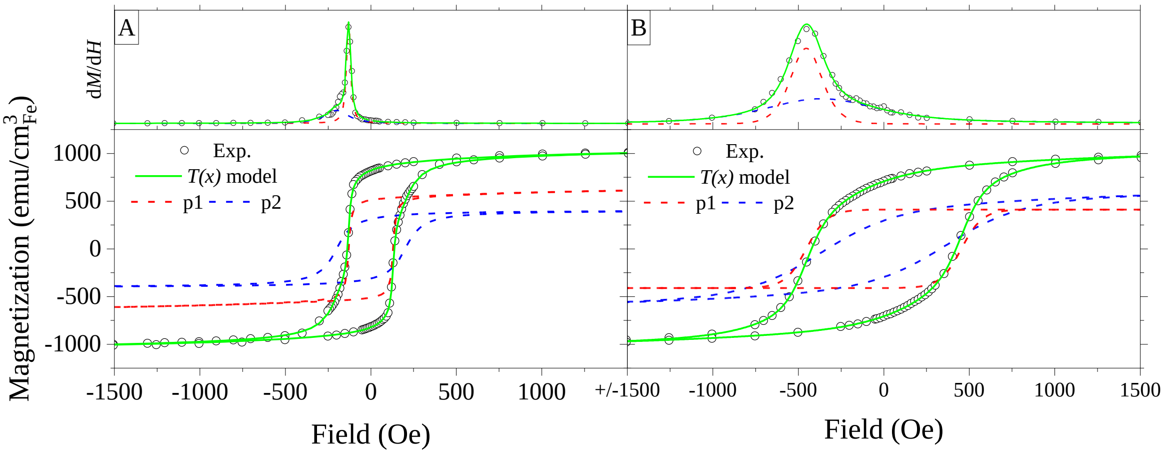

Figure S1. Derivative of the upper branch of hysteresis (top graphs) and modeled magnetization curves (bottom graphs) for as-prepared (A) and annealed (B) thin iron film deposited on ATiO with 50 nm pores. Calculated magnetic phases are shown with dashed lines.

Figure S2. displays the amplification factor of the coercive field resulting from the nanopatterning and annealing of the Ti/ATiO/Fe samples. The values are determined at 10 K and normalized to the values of H_c of sample S0. Enhancement is shown as a function of the inverse difference between the average distance between centers of the pores D_c and average inner pore diameters D_p.

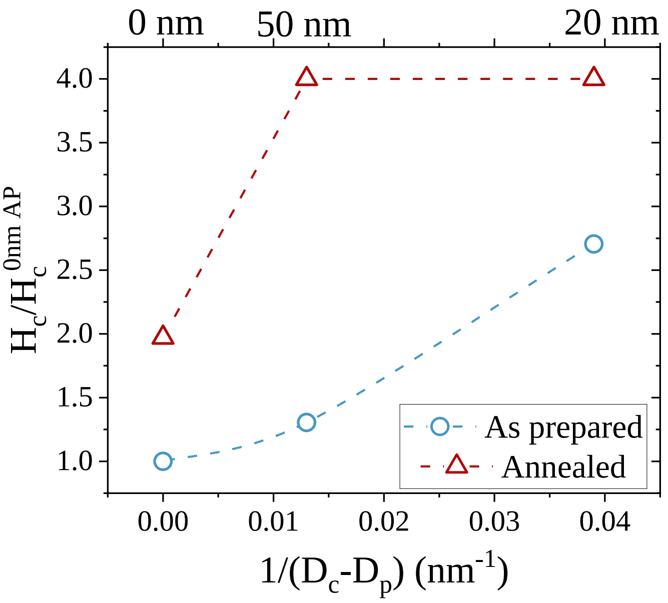

Figure S2. Enhancement of coercive field for nanopatterned samples.

Figure S3. shows X-ray photoelectron spectra of iron measured at the ATiO/Fe interface for the sample S0. Measurements reveal strong oxidation at the boundary between anodized titanium oxide and the iron layer.

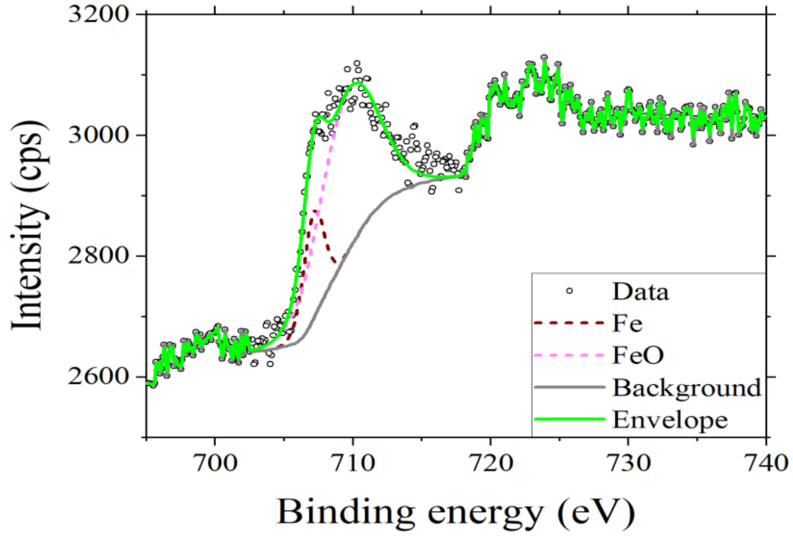

Figure S3. XPS measurement made at Fe/ATiO interface for as-prepared mesoporous sample.

The values of the effective anisotropy constant K_{eff} are shown in Figure S4 and were calculated according to the expression:

$$K_{eff} = \left(\int_{-Ha}^{Ha} M(H)_{IP}^{\uparrow} dH - \int_{-Ha}^{Ha} M(H)_{OOP}^{\uparrow} dH \right) /2,$$

where $M(H)^{\uparrow}$ is an upper branch of the magnetization curve for in-plane (IP) or out-of-plane (OOP) (see Figure S4) geometry and H is an applied magnetic field changing in the range of the anisotropy field $\pm H_a$ defined as saturation field for hard magnetic direction.

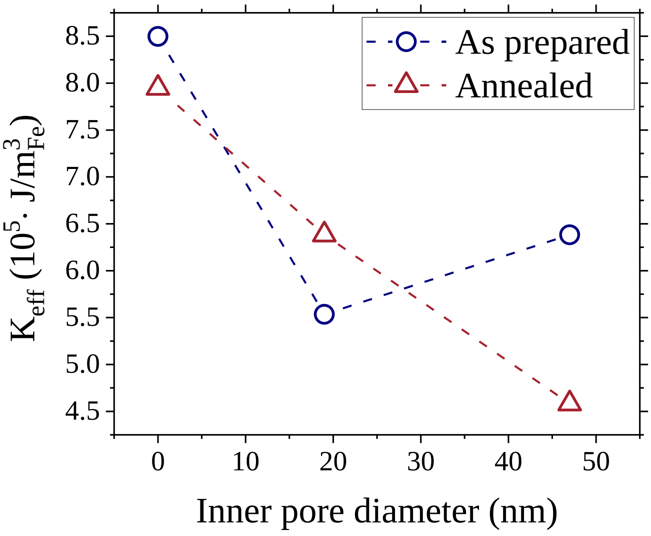

Figure S4. Effective anisotropy constant as a function of inner pore diameter for as-prepared and annealed Fe/ATiO samples.

Hysteresis loops of sample S0 before and after annealing measured for two geometries, where a magnetic field is applied parallel (in-plane) or perpendicular (out-of-plane) to the surface of samples (see Figure S5). Measurements were performed at 10 K.

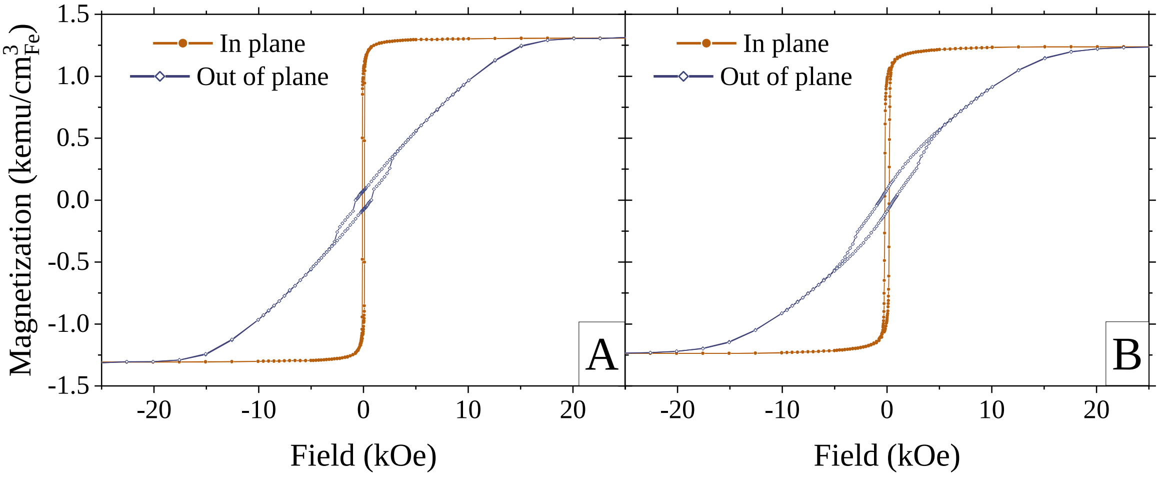

Figure S5. Magnetization curves for as-prepared (A) and annealed (B) thin iron film deposited on mesoporous ATiO at in-plane and out-of-plane geometry.
